# Operative treatment of intra-articular calcaneal fractures with calcaneal plates and its complications

**DOI:** 10.4103/0019-5413.49388

**Published:** 2009

**Authors:** Vaclav Rak, Daniel Ira, Michal Masek

**Affiliations:** Department of Trauma Surgery, University Hospital Brno, Czech Republic

**Keywords:** Calcaneal plate, intra-articular calcaneal fracture, lateral extensile approach

## Abstract

**Background:**

In a retrospective study we analysed intra-articular calcaneal fracture treatment by comparing results and complications related to fracture stabilization with nonlocking calcaneal plates and locking compression plates.

**Materials and Methods:**

We performed 76 osteosynthesis (67 patients) of intra-articular calcaneal fractures using the standard extended lateral approach from February 2004 to October 2007. Forty-two operations using nonlocking calcaneal plates (group A) were performed during the first three years, and 34 calcaneal fractures were stabilized using locking compression plates (group B) in 2007. In the Sanders type IV fractures, reconstruction of the calcaneal shape was attempted. Depending on the type of late complication, we performed subtalar arthroscopy in six cases, arthroscopically assisted subtalar distraction bone block arthrodesis in six cases, and plate removal with lateral-wall decompression in five cases. The patients were evaluated by the AOFAS Ankle-Hindfoot Scale.

**Results:**

Wound healing complications were 7/42 (17%) in group A and 1/34 (3%) in group B. No patient had deep osseous infection or foot rebound compartment syndrome. Preoperative size of Böhler's angle correlated with postoperative clinical results in both groups. There were no late complications necessitating corrective procedure or arthroscopy until December 2008 in Group B. All late complications ccurred in Group A. The overall results according to the AOFAS Ankle Hindfoot Scale were good or excellent in 23/42 (55%) in group A and in 30/34 (85%) in group B.

**Conclusion:**

Open reduction and internal fixation of intra-articular calcaneal fractures has become a standard surgical method. Fewer complications and better results related to treatment with locking compression plates confirmed in comparison to nonlocking ones were noted for all Sanders types of intra-articular calcaneal fractures. Age and Sanders type IV fractures are not considered to be the contraindications to surgery.

## INTRODUCTION

**Figure 1:**
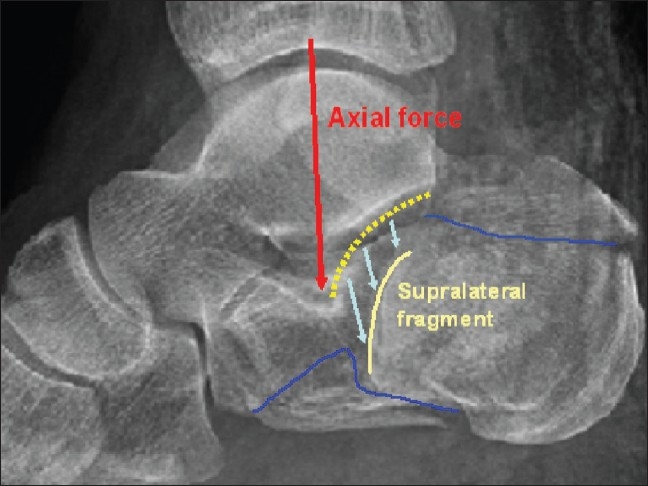
Direction of axial force to the vertex of the Gissane's angle and formation of the typical supralateral fragment due to articular facet break-through. Blue line – secondary fracture line

Calcaneal fractures are relatively rare injuries, with reported occurrence of 2% of all fractures. The intra-articular types constitute to 75% of calcaneal fractures.^1^,^2^ These fractures originate due to axial force in axis of crus when processus lateralis tali impacts as a hammer on the area of Gissane's angle (G-angle) vertex and thus breaks through the posterior articular facet [Figure 1]. The functional sequelae of the foot are serious, and therapy is economically demanding.^1^–^3^ The treatment strategy of calcaneal fractures and their complications have been extensively reported. Despite it being a subject of debate, open reduction and internal fixation of displaced fractures is generally preferred.^1^–^25^

Plate osteosynthesis of the intra-articular fractures is a standard treatment method, but it has potential complications such as poor wound healing and infection. The complications of wound healing occur because of not enough precisely performed incisions. Calcaneal shape restoration by means of open reduction internal fixation (ORIF) or primary subtalar arthrodesis if needed is mandatory prevention of late complications such as malposition, flattening of the longitudinal arch, anterior ankle impingement syndrome, lateral impingement syndrome, and axial malalignment of the hind foot^1^,^3^,^5^,^8^,^12^,^16^,^21^,^26^. The locking compression plate (LCP) has improved the functional results, limited the indications for bone grafting, and shortened the treatment. The purpose of our study is to compare the functional results and complications of fractures treated with calcaneal nonlocking plates and locking compression plates.

## MATERIALS AND METHODS

76 intra-articular calcaneal fractures in 67 patients (9 simultaneous bilateral fractures) were treated by means of open reduction and internal plate fixation from February 2004 to October 2007.

The most frequent mechanism of injury was fall from height, occurring as an isolated fracture of lower limb. There were 57 males and 10 females with an average age of 44 years (range 23–73 years), median 43). Three patients suffered open fractures (grade I, two fractures; and grade II, one fracture-Tscherne classification) with the wounds from the medial side of the calcaneal area. Group A (n = 42) includes patients treated with nonlocking calcaneal plates for the first three years, and group B (n = 34) includes patients operated in 2007 with LCP [Table 1]. In two cases, we performed arthroscopically assisted osteosynthesis (both in group B). When nonlocking plates were used (group A), we filled the defect with bone graft in 79% (n =33) cases (Sanders type II, 13 fractures; Sanders type III, 16 fractures Sanders type IV, 4 fractures). In group B, bone grafting was performed in 9% cases (n=3), only for Sanders type IV fractures.

**Table 1:** Characteristics of studied groups (Calcaneal fracture study: 76 operations, 67 patients)

Group A	Group B
2004-2006	2007
42 osteosyntheses	34 osteosyntheses
Nonlocking calcaneal plates	Locking compression plates
Bone grafting - 82%	Bone grafting - 9%

Sanders computed tomography (CT) scan classification is based on articular fracture lines of the posterior articular calcaneal facet (A, lateral; B, central; C, medial), and the severity of the fracture (Sanders types I–IV) depends on the number of lines and their courses.

Dislocated calcaneal fractures (posterior articular facet step off more than 2 mm, significant shortening, loss of height, and widening of the calcaneus, i.e., Böhler's and Gissane's angle decrease, valgus deviation >10°, varus deviation >5°) of Sanders type II, III, and IV were included in operative treatment. Serious medical ailments and poor soft tissue envelope were contraindications to surgery. Age was a relative contraindication. Biological state of patient was crucial for treatment decision. We operated patients with Sanders type II–IV fractures [Table 2]. The primary subtalar arthrodesis with calcaneal osteosynthesis required when the articular surface was nonreducible (only Sanders type IV injuries n=5). In all these cases, we performed primary osteosynthesis with restoration of the calcaneal shortening, loss of height, and broadening. Patients who underwent subtalar arthrodesis as a primary procedure after the injury were not included in our study. In group A, six patients underwent subtalar arthrodesis as a secondary procedure on average 13 months (range, 8–20) after osteosynthesis due to clinically significant signs of subtalar arthrosis correlating with X-ray findings.

**Table 2:** Fracture distribution according to Sanders classification

Sanders classification	Group A	Group B
Type I nondisplaced fractures	–	–
Type II two-part fractures (split)	19	10
Type III three-part fractures	18	18
Type IV four-part fractures, highly comminuted	5	6

### Operative procedure

**Figure 2:**
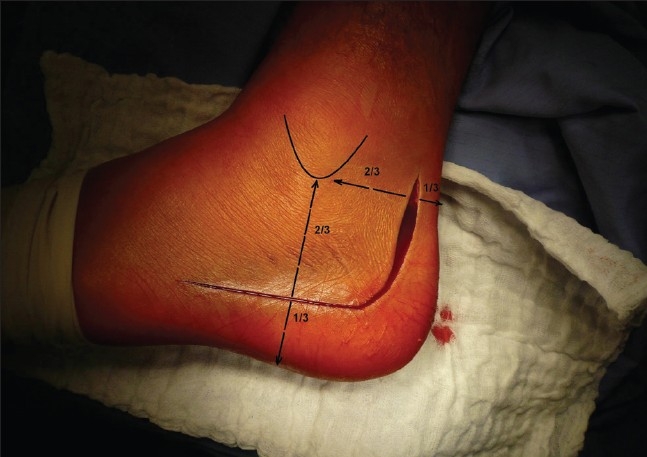
Clinical photograph showing standard extended lateral approach

**Figure 3:**
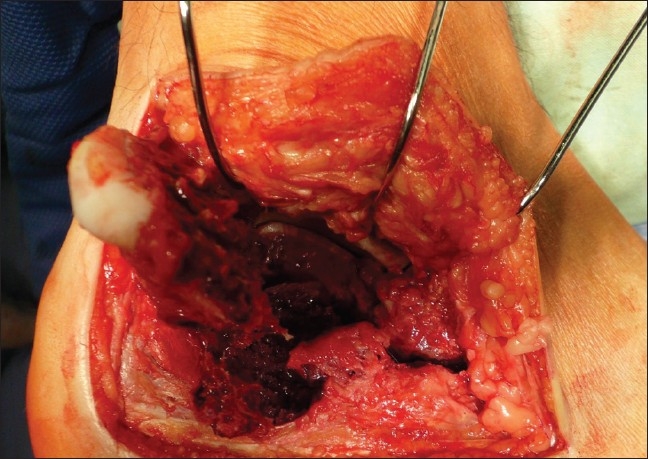
Intraoperative photograph showing typical supralateral fragment

**Figure 4:**
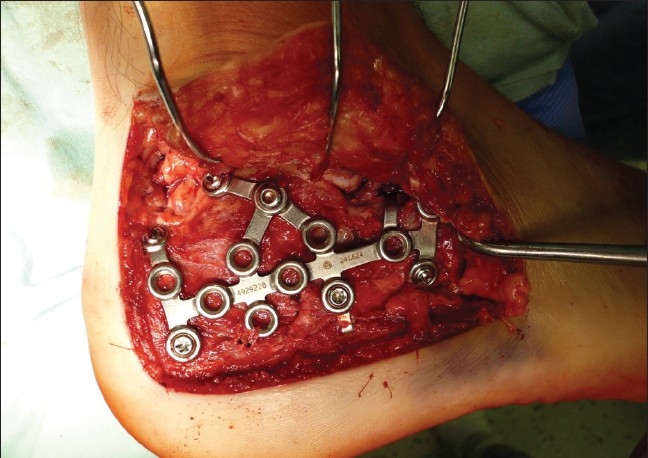
Intraoperative photograph showing restoration of Gissane's angle and implanting LC plate

**Figure 5:**
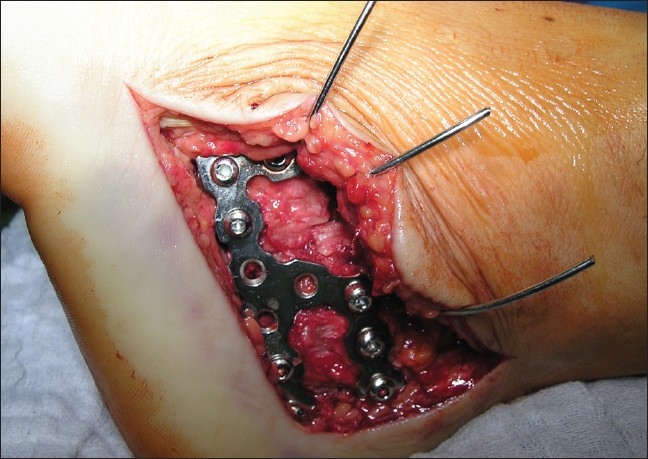
Intraoperative photograph showing nonlocking calcaneal plate

We usually operated from the seventh to tenth day after injury (range 2–21 days, average 8.0 days), when the soft tissue edema decreased and there were positive wrinkles on hind foot soft tissues. The surgery was performed under tourniquet, with the patient placed in lateral decubitus position, under intraoperative fluoroscopy control (Broden's views). The standard extended lateral approach with L-shaped incision type Seattle and no-touch technique were used [Figure 2]. Posterior and anterior calcaneal facets reconstruction including the “bridging” in Gissane's angle, Böhler's angle with calcaneal height, width, length restoration, and no varus–valgus deviation were main goals of open reduction [Figures 3–5]. Depending on fracture type, size of the defect, and type of calcaneal plate, we filled up the defect with autogenous cancellous bone [Table 1]. All operations were done by three surgeons with experience of implanting calcaneal plates from previous years (1998–2004).

**Figure 6:**
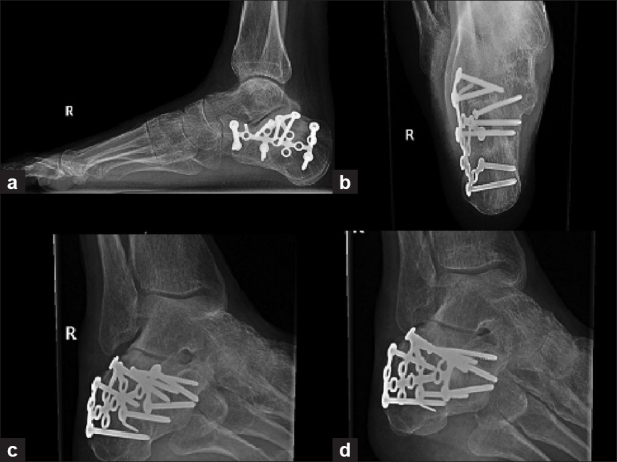
Lateral (a) and axial (b) view of calceneum and Broden's oblique views (c,d) shows calcaneal plates fixations.

After the surgery, the compressive elastic bandage was given. Range of motion exercises began immediately on the first postoperative day. The progressive weight-bearing was started after 8 weeks, initially with 30% of their weight. Patients were allowed full weight-bearing after 12 weeks, and 2 weeks later in case of bilateral calcaneal fractures provided that reduced and stabilized fracture position stayed unchanged and signs of bone healing were present. When LCP had been applied, we started up progressive weight-bearing two weeks earlier, because of better stability of locked screws in plate. Hardware removal was considered unnecessary unless there were complications. X-ray imaging, including Broden's oblique views, were obtained on the 4^th^, 8^th^, and 12^th^ week, and then 6^th^, 12^th^, and 24^th^ month after the surgery [Figure 6]. CT scans were carried out at one-year follow-up.

**Figure 7:**
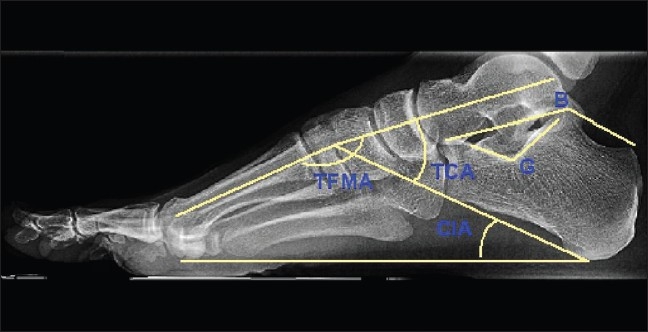
X-ray foot and ankle (lateral view) showing measured and assessed angles. B - Böhler's angle (20-40°), G - Gissane's angle (120-145°), TCA – talocalcaneal angle (25-50°), TFMA – talus first metatarsal angle (0 – 10°), CIA – calcaneal inclination angle (20 – 25°)

The patients were followed up for 12–32 months after the surgery (group A. 20–32 months; group B, 12–17 months). Assessments and measurements were done 3 months after the surgery, and then one (Group A and B) and two years (Group A) after the injury. We analyzed the radiographs and CT scans one year after the operations. The late complications and patients' satisfaction were evaluated at regular outpatient examinations. On the radiographs and CT scans, we evaluated Böhler's, Gissane's, talocalcaneal angle (TCA), talus first metatarsal angle (TFMA), and calcaneal inclination angle (CIA) [Figure 7].

**Figure 8:**
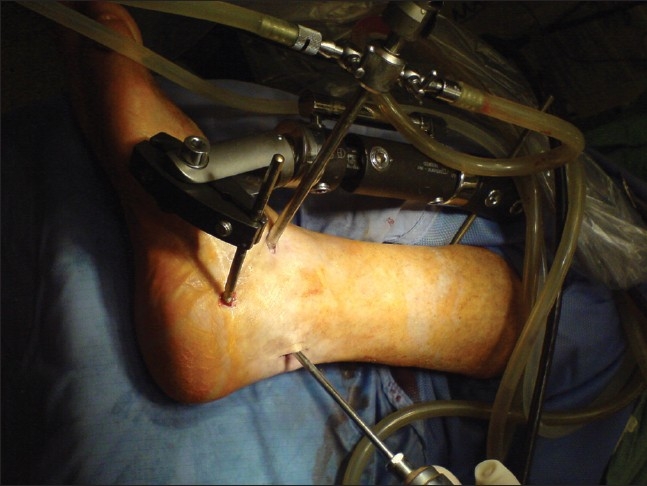
Crinical photograph of arthroscopically-assisted subtalar arthrodesis with distraction device

**Figure 9:**
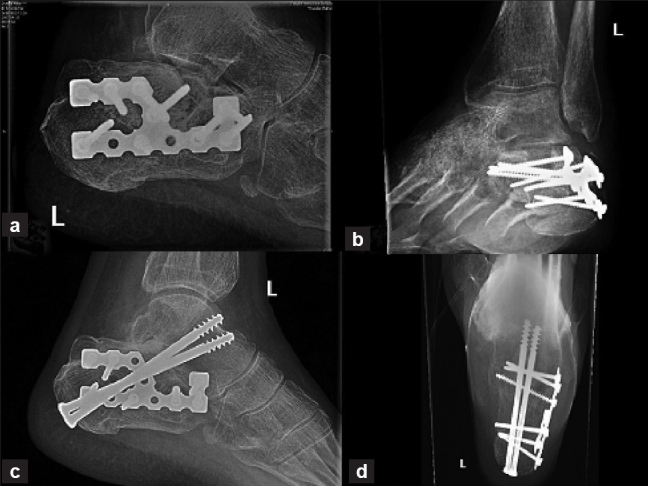
X-rays of ankle joint (lateral and oblique views) showing imperfect reconstruction of cancaneal shape, decreased bohler's angle (a) and non reduced posterior facet (b), which lead to post traumatic arthritis pain. X-ray of ankle joint (lateral and anteroposterior view) showing subsequent arthroscopically assisted reconstructive isolated subtalar distraction tricortical arthrodesis (c, d).

For the evaluation of posterior articular facet defect or congruency, Sanders criteria for subtalar joint reduction and, for posttraumatic posterior facet degenerative changes, Allmachers Arthrosis Rating Scale were used [Table 3]. Incidence and severity of posttraumatic subtalar arthrosis depends on the fracture type, calcaneal shape, and position after the osteosynthesis, chondral injury of the subtalar joint, and articular facet congruency. Clinically significant complaints (Sanders criteria for subtalar joint reduction grades III and IV and Allmachers Arthrosis Rating Scale grades II to V) occurred in 13 cases only in Group A. Arthroscopically assisted isolated subtalar distraction tricortical arthrodesis (ISDTA) was performed through posterolateral and anterolateral portals using bone grafts from the iliac crest. The plate was left in place, and bone graft and bones were fixed with 6.5-mm cannulated cancellous screws [Figures 8 and 9]. The limb was fixed in non–weight-bearing under knee plaster cast for the first 6 weeks after the surgery, and partial weight-bearing in special orthosis for additional six weeks was allowed.

**Table 3:** Evaluation criteria

Criteria for roentgenographic subtalar joint reduction (Sanders 1993)	Arthrosis rating scale (Allmacher 2006)
Anatomic, no articular incongruity whatsoever	0 Normal
Near-anatomic, < 3 mm of articular incongruity or gapping between fragments	I Osteophyte only
Approximate, 3-5 mm of articular incongruity or gapping between fragments	II Osteophyte with subchondral cysts and normal joint space
Failure, > 5 mm of articular incongruity or gapping between fragments	III Grade II + mild narrowing of the joint space
	IV Severe joint space narrowing
	V Fused

Patients in both groups were evaluated by a unified scoring system AOFAS Clinical Rating System, the Ankle Hindfoot Scale for calcaneal area (100 points total, 90–100 points, excellent; 80–89 points, good; 70–79 points, fair, less than 70, poor).

## RESULTS

### Wound healing

Sixty of all 76 wounds (79%) healed primarily, superficial defects (wound edge necrosis, oedema blisters) occurred in 8 (10.5%) cases, and serious wound healing complications occurred in 8 (10.5%) cases. Superficial defects were trivial and had no influence on fracture healing, and thus they were excluded from the wound healing complications deep defects, when the plate had to be removed for subsequent successful healing process, were noticed in six cases. Time of hardware removal because of these complications varied from 7 weeks to 4 months after the osteosynthesis. All defects healed conservatively; no plastic surgery was needed, and no fragments redislocations occurred after forced plate removal. No patient had deep osseous infection or rebound compartment syndrome, and no limb amputation was necessary to perform [Table 4].

**Table 4:** Soft tissue complications

Soft tissue coverage healing	No. of operations		Ratio Group	Total
			
	Group A	Group B	A + B	
Primary healing	31 (74)	29 (85)	(79)	(89.5)
Superficial defect	4 (9)	4 (12)	(10.5)	
Severe defect of the soft tissue envelope	2 (5)	0	(2.5)	(10.5)
Severe defect with plate removal	5 (12)	1 (3)	8	

Figures in parentheses are in percentages

### AOFAS-Ankle-Hindfoot Scale

In group A (n = 42), we achieved excellent to good results in 54.5 %, Sanders type II – 9/19 (47%), III – 13/18 (72%), IV – 1/5 (20%) and in group B (n = 34) in 85%, Sanders type II – 9/10 (90%), III – 15/18 (83%), IV – 5/6 (83%). In both groups together, there were 24/76 (32%) of patients with excellent results, 28/76 (37%) with good results, 14/76 (18%) with fair results, and 10/76 (13%) with poor results. In group B, there was just one patient with a poor result and four patients with fair results [Tables 5 and 6].

**Table 5:** AOFAS score versus Sanders classification - Group A

Sanders type	Excellent	Good	Fair	Poor	Total
II	4	5	7	3	19
III	5	8	2	3	18
IV	-	1	1	3	5
Total	9 (21.5)	14 (33)	10 (24)	9 (21.5)	42

Figures in parentheses are in percentages

**Table 6:** AOFAS score versus Sanders classification - Group B

Sanders type	Excellent	Good	Fair	Poor	Total
II	7	2	1	–	10
III	8	7	2	1	18
IV	–	5	1	–	6
Total	15 (44)	14 (41)	4 (12)	1 (3)	34

Figures in parentheses are in percentages

### Group A

Table 7 shows assessments of particular measured angles in group A. Thirteen patients with clinically significant signs of subtalar arthrosis (Allmacher grade II-IV) were evaluated and underwent another surgical procedure. Six times, we performed subtalar arthroscopy with joint debridement and arthrofibrosis resection, other six patients underwent arthroscopic-assisted isolated subtalar distraction tricortical arthrodesis (ISDTA) due to serious persisting subjective complaints, calcaneal malposition, signs of lateral and tibiotalar impingement, and decrease in talocalcaneal angle (TCA) and calcaneal inclination angle (CIA). We achieved satisfactory clinical results using both techniques mentioned above.

**Table 7:** Preoperative and postoperative average angle assessment depending on fracture type - Group A

Fracture type - Sanders classification	II (n=19)			III (n=18)			IV (n=5)		
			
Assessed angle	N	Aver.	M	N	Aver.	M	N	Aver.	M
B angle									
Bef. OS	19	1.8	5.0	18	9.3	12.0	5	1.0	4.0
After OS	19	25.6	27.4	18	28.2	28.0	5	21.3	23.0
After 1 yr.	19	23.8	26.4	18	25.3	26.8	5	18.4	22.0
After 2 yr.	13	22.8	28.0	13	24.5	23.0	0		
G angle									
After OS	19	122.6	121.9	18	125.7	122.8	5	127.7	132.0
After 1 yr.	19	120.1	121.3	18	121.4	120.0	5	126.2	130.8
After 2 yr.	13	119.3	120.0	13	116.0	118.0	0		
TCA angle									
After OS	19	37.3	38.4	18	38.1	38.0	5	32.0	31.0
After 1 yr.	19	35.5	36.9	18	36.4	35.0	5	30.6	28.0
After 2 yr.	13	35.0	35.0	13	34.4	34.0	5		
TFMA angle									
After OS	19	7.5	4.4	18	5.7	5.0	5	8.1	7.0
After 1 yr.	19	8.6	7.0	18	6.2	5.8	5	7.3	7.0
After 2 yr.	13	7.3	6.0	13	5.9	6.0	0		
CIA angle									
After OS	19	23.6	23.0	18	23.3	23.0	5	24.9	23.0
After 1 yr.	19	21.1	20.4	18	20.3	20.0	5	19.9	18.0
After 2 yr.	13	20.1	20.0	13	20.1	20.0	0		

N - number of patients, M - median, bef. OS - before osteosynthesis, yr. - year, OS - osteosynthesis, Aver. - average

Sanders type IV fractures were not assessed after two years, 4 patients underwent isolated subtalar distraction tricortical arthrodesis

Signs of the lateral and tibiotalar impingement syndrome occurred in eight cases. Two patients with lateral impingement syndrome were resolved by plate removal, lateral wall decompression, and lateral wall ostectomy; in one case, plate removal alone was sufficient.

There were three patients who had already undergone ISDTA and suffered from both previous syndromes. Subsequently, plates were removed and lateral wall ostectomy was performed [Table 8].

**Table 8:** Calcaneal fracture sequelae - Group A

Late complications Group A	No. of operations	Ratio%	Subsequent procedures	No. of operations	Ratio%
Subtalar arthritis - grade II-IV	13	31	ASK	6	14,3
			ISDTA	6	14,3
Lateral, tibiotalar impingement syndrome	8	19	PR, LWE	5	12
Dysesthesia	3	7,1			
Neurovascular complications	0	0			

PR - plate removal, LWE - lateral-wall ostectomy, ASK - subtalar arthroscopy, ISDTA -isolated subtalar distraction tricortical arthrodesis.

We analyzed the results of group A and we found out the following statistically significant conclusions (final *P* values were compared with the significance level at 0.05).

Satisfaction of the patients with Böhler's angle larger or equal to 20° (preoperatively) was statistically significant and higher than those with Böhler's angle smaller than 20° (*P* = 0.037).

Patients with preoperative Böhler's angle larger or equal to 20° (n = 3) achieved excellent to good results in three cases (100%); patients with preoperative Böhler's angle smaller than 20° (n=39) achieved excellent to good results in 20 cases (51%).

Patients with Böhler's angle (preoperatively) smaller than 20° (*P* = 0.001) had a higher rate of the postoperative posterior facet defects than in patients with B-angle equal to 20° or larger.

### Group B

Because of shorter time from the operation and excellent postoperative achievements with LCP implants, we did not notice any late complication indicated to the corrective procedure in this group. Two-year follow-up assessments were not done.

Satisfaction of the patients with Böhler's angle larger or equal to 20° (preoperatively) was statistically significant and higher than those with Böhler's angle smaller than 20° (*P* = 0.024).

Patients with preoperative Böhler's angle larger or equal to 20 (n = 5) achieved excellent to good results in five cases (100%); patients with preoperative Böhler's angle smaller than 20° (n = 29) achieved excellent to good results in 23 cases (79%).

Table 9 shows assessments of the particular measured angles.

**Table 9:** Preoperative and postoperative average angle assessment depending on fracture type - Group B

Fracture type - Sanders classification	II (n=10)			III (n=18)			IV (n=6)		
			
Assessed angle	N	Aver.	M	N	Aver.	M	N	Aver.	M
B angle									
bef. OS	10	8.7	11.0	18	6.6	6.0	6	1.0	1.5
After 1 yr.	10	31.2	30.5	18	31.9	33.0	6	34.3	36.0
G angle									
After 1 yr.	10	123.4	123.0	18	122.4	121.0	6	122.3	121.0
TCA angle									
After 1 yr.	10	39.7	38.5	18	38.9	38.5	6	39.5	39.0
TFMA angle									
After 1 yr.	10	7.4	7.0	18	4.7	4.0	6	5.3	3.0
CIA angle									
After 1 yr.	10	25.7	24.5	18	22.2	23.0	6	23.7	26.5

N - number of patients, M - median, bef. OS - before osteosynthesis, OS - osteosynthesis, Aver. - average, yr - year

## DISCUSSION

In the last decade, open reduction and internal plate fixation of dislocated intra-articular calcaneal fractures has become a standard surgical method with low complication rate and better quality of life after the surgery.^1^–^25^ The method has been improved by implanting locking compression plates, the osteosynthesis is more stable, enables earlier weight-bearing, and bone grafting is rarely necessary.^5^,^12^,^20^,^23^ Brauers cost-effectiveness analysis of surgery versus conservative treatment for intra-articular calcaneal fractures showed economical advantage of ORIF.^2^ In 2004, the comparison of five multicentric studies regarding conservative and operative treatment (issued in medline between January 1999 and March 2004) was published. Results of these comparative studies also favoured operative treatment over conservative one. Most of the conservatively treated patients underwent arthrodesis procedure. Poorer health and social prognosis is related to males, heavy workers, patients with B-angle smaller than 0°, bilateral fractures, and Sanders type IV fractures.^15^

Conclusions of Bajammal who analyzed 20 publications dealing with operative vs. conservative treatment showed significant benefits of surgical therapy for females, young males, patients with lighter workload, and patients with initially high B-angle or with simple, minimally dislocated fractures, whereas older males and those who have an occupation involving heavy workload had benefited from the conservative therapy.^27^ Buckley (analysis of 559 calcaneal fractures)^13^ and Tufescu reported similar findings^25^ and both definitely recommended operative treatment.

In our study, we confirmed the benefits of LCP implanting over nonlocking calcaneal plates for all Sanders types of intra-articular fractures. The rate of wound healing complications, especially the rate of severe defects was lower in group B. Patients in group B reached better clinical results according to the AOFAS Hind Foot Scale. The most significant increase of satisfied patients was noticed in group of Sanders IV type fractures. Clinical results of Sanders type II and III fractures operated with locking compression plates were better than those stabilized with nonlocking calcaneal plates. High percentage of operated fractures that required bone grafting in group A showed another advantage of locking compression plates. In group B, only huge bone defects after reduction (Sanders type IV fractures) had to be filled with bone graft.

Herscovici proved that there were no significant risks of wound healing for patients older than 65^17^. In our series the age was not a contraindication to operation. We operated five patients older than 65; four wounds of five healed by primary intention and according to the AOFAS Ankle Hindfoot Scale all patients' results were evaluated as good or excellent.

Rate of wound healing complications that achieved 10.5% in our study is comparable with the results published in literature of the last decade. Superficial wound complications, when microbial agens were proved occurred in 6.3%. In 2004, Zwipp presented one of the biggest studies of calcaneal fracture treatment: 496 patients with 553 fractures (90% treated operatively; 95% lateral approach, 1.5% bilateral approach, 1% medial approach, 2.2% percutaneous miniinvasive osteosynthesis, and primary fusion in 0.3%). He used intra-operative open arthroscopy to control articular joint reduction. In this study the implanting of LCP enabled to decrease the use of bone grafting from 53% (nonlocking plates) to 3.8% (LCP). In the group of 453 fractures treated by ORIF apical wound necrosis was noticed in 6.7%, evacuated hematoma in 4.7%, soft tissue infection in 4.3% and bone infection in 2.2%. Limb amputation was not performed but compartment syndrome occurred in 2.2%. Good or excellent results were achieved by 72% of patients.^12^ Sander's analysis of soft tissue and late complications and its relevance to type II to IV fractures proved that operation demandingness, rate of complications, and patients' dissatisfaction grow with fracture severity. In this study of 120 patients, one limb amputation had to be performed and wound healing complication rate achieved 5%.^6^–^8^

We operated within first two weeks after injury because the surgery in the third week from injury is burdened with higher percentage of soft tissue healing complications and ORIF performed with more than three weeks delay is not recommended.^1^,^4^,^8^,^12^,^16^,^26^

In our analysis, we confirmed correlation between the Böhler's angle size and patient satisfaction in groups A and B, as well as dependence of articular joint incongruence and the subsequent subtalar arthrosis on diminishing B-angle (only group A).This fact, proved and verified by a lot of other authors, confirms the role of Böhler's angle size as a predictive factor for subsequent late complications.^13^,^16^ Loucks in his prospective randomized study pointed out that initial negative size of Böhler's angle negatively influences postoperative results irrespective of therapy choice.^29^

In our opinion, there is no indication for bone grafting when LCP are used except for the Sanders IV type injuries with huge defects after the reduction. Longino had compared postoperative radiological and clinical results of LCP osteosynthesis with and without bone grafting and did not find any significant difference.^20^ In accordance with other authors, by intra-articular calcaneal fracture treatment, we emphasize right operation timing, knowledge in anatomy, sufficient size of lateral approach, no-touch technique, perfect posterior facet fragment reduction in subtalar joint, restoration of calcaneal height, width and length with calcaneal-cuboid joint revision. Inaccurate reduction and malposition leads to persistent complaints. In five cases of comminuted Sanders type IV fractures with nonreducible subtalar joint facet (these fractures were not included in the study) we performed primary arthrodesis using calcaneal plate with calcaneal shape restoration. Nonoperative treatment or poor intra-operative calcaneal shape restorations of these injuries results in irreparable late sequelae and patient complaints.^1^,^4^,^8^,^12^,^14^,^16^,^30^

Certain types of fractures (Sanders type II and II) can be indicated to miniinvasive osteosynthesis with arthroscopic assistance using screws and K-wires.^4^,^12^,^16^,^23^,^31^,^32^

Six patients after open surgery with subsequent clinically manifested subtalar arthrosis underwent subtalar arthroscopic debridement with arthrofibrosis resection.

We operated six patients, performing ISDTA in accordance with other authors' recommendations. This technique was first published by Carr in 1988.^30^ Carro documented posterior and lateral arthroscopic approach for ISDTA in contrast to only lateral approaches that were previously used.^33^ We used this approach for ISDTA, too. In literature, recommendations for ISDTA performance prevails over those for arthrodesis in situ with lateral wall decompression.^34^–^36^ Rammelts recommends performing subsequent arthrodesis within 2 years after the injury after careful patient's preparation and education. He prefers this method because of excellent results in his study (31 patients, 93.6% satisfied patients according to the AOFAS Ankle Hindfoot Scale).^4^ Review of experience with different types of symptomatic non-union after calcaneal fracture was published by Molloy in 2007.^35^

**Figure 10:**
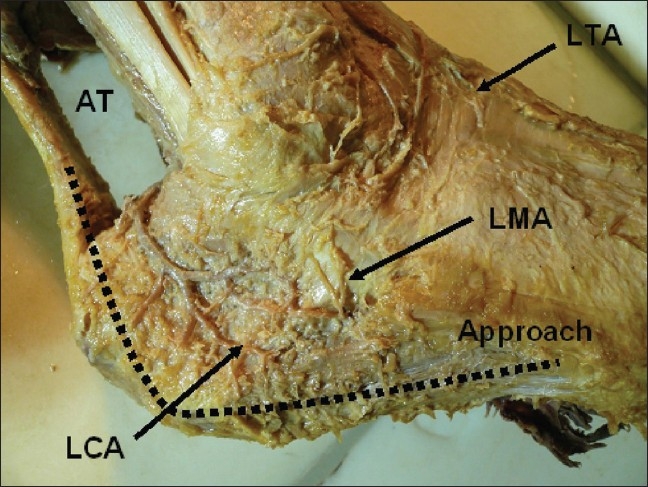
Cadaveric photograph shows soft tissue vascularization of the lateral wall of the hind foot on cadaver. LCA - lateral calcaneal artery, LTA - ventrolateral tarsal arteries, LMA - lateral malleolar artery, AT – Achilles tendon

Knowledge in anatomy especially anatomy of the lateral hindfoot vascularity with its standard architecture is important prevention of wound healing complications. Our experimental study supported the importance of precisely performed L shaped incision that respects the blood supply. The blood supply of lateral hind foot had standard course and forms arterial arch. The course of arch defined the outline of the lateral extended approach incision for calcaneal fractures that lied fairly close to lateral outline of this vascular arcade. Inaccurate making of incision with injuring the vessels result inevitably in serious ischemic complications [Figure 10].^22^ Andermahr's dissection study of 10 and 13 cadavers verified the standard course of the great arterial arch. Lateral impaction of the fragments, inappropriate operative incision and fracture immobilization with lateral splint (that may lead to aseptic necrosis of the fracture fragments) can result in lateral calcaneal artery injury.^37^,^38^ Borrelli in his study of 24 cadavers published similar results and emphasized the importance of no touch technique and arterial arch preserving when operative incision were performed.^39^

ORIF with open arthroscopy assistance and 3D X-ray imaging have become standard procedures in current orthopaedics. When LCP is implanted, there is no need for bone grafting. Arhroscopically-assisted osteosynthesis is a current trend for treatment of particular fracture types. Age is not a contraindication. AOFAS clinical rating system the Ankle Hindfoot Scale for calcaneal area is a standard scoring system for foot function evaluation.^28^ Using this standard scoring system that takes into account subjective and objective assessments enables to achieve relevant results and comparisons of different patients' studies. Finally, one has to mention optimistic findings of Melcher who followed up patients operated by ORIF 3 and 10 years after the surgery. In his study, subjective and objective results assessed after ten years were better than those achieved in a 3-year follow-up.^40^

Well timed open reduction and internal fixation with LCP in an indicated case, respecting soft tissue envelope with or without arthroscopic assistance and early rehabilitation lead to therapeutic success. Standard operation incisions with sufficient access approach are precautions to soft tissue coverage healing complications. In general these fractures are operated with some delay. Considering the rare incidence of these fractures, need of special hardware equipment and relevant experience, the primary management of these injuries as well as complication treatment should be centred in specialized departments of orthopaedics or traumatolgy.
